# Mental Stress in Atopic Dermatitis – Neuronal Plasticity and the Cholinergic System Are Affected in Atopic Dermatitis and in Response to Acute Experimental Mental Stress in a Randomized Controlled Pilot Study

**DOI:** 10.1371/journal.pone.0113552

**Published:** 2014-12-02

**Authors:** Eva Milena Johanne Peters, Anna Michenko, Jörg Kupfer, Wolfgang Kummer, Silke Wiegand, Volker Niemeier, Nikolay Potekaev, Andrey Lvov, Uwe Gieler

**Affiliations:** 1 Psychoneuroimmunology Laboratory, Joint appointment a) Center for Internal Medicine and Dermatology, Universitätsmedizin-Charité, Berlin, and b) Department of Psychosomatic Medicine, Justus-Liebig-University, Giessen, Germany; 2 Department of Dermatology, I. M. Sechenov Moscow Medical University, Moscow, Russia; 3 Institute of Medical Psychology, Justus-Liebig University, Giessen, Germany; 4 Institute of Anatomy and Cell Biology, Justus-Liebig-University, Giessen, Germany; 5 Department of Psychosomatics and Psychotherapy, Justus-Liebig-University, Giessen, Germany; 6 Department of Dermatology, University Hospital Giessen, Giessen, Germany; 7 Moscow Scientific and Practical Center of Dermatovenereology and Cosmetology, Moscow, Russia; Ohio State University, United States of America

## Abstract

**Rationale:**

In mouse models for atopic dermatitis (AD) hypothalamus pituitary adrenal axis (HPA) dysfunction and neuropeptide-dependent neurogenic inflammation explain stress-aggravated flares to some extent. Lately, cholinergic signaling has emerged as a link between innate and adaptive immunity as well as stress responses in chronic inflammatory diseases. Here we aim to determine in humans the impact of acute stress on neuro-immune interaction as well as on the non-neuronal cholinergic system (NNCS).

**Methods:**

Skin biopsies were obtained from 22 individuals (AD patients and matched healthy control subjects) before and after the Trier social stress test (TSST). To assess neuro-immune interaction, nerve fiber (NF)-density, NF-mast cell contacts and mast cell activation were determined by immunohistomorphometry. To evaluate NNCS effects, expression of secreted mammal Ly-6/urokinase-type plasminogen activator receptor-related protein (SLURP) 1 and 2 (endogenous nicotinic acetylcholine receptor ligands) and their main corresponding receptors were assessed by quantitative RT-PCR.

**Results:**

With respect to neuro-immune interaction we found higher numbers of NGF+ dermal NF in lesional compared to non-lesional AD but lower numbers of Gap43+ growing NF at baseline. Mast cell-NF contacts correlated with SCORAD and itch in lesional skin. With respect to the NNCS, nicotinic acetylcholine receptor α7 (α7nAChR) mRNA was significantly lower in lesional AD skin at baseline. After TSST, PGP 9.5+ NF numbers dropped in lesional AD as did their contacts with mast cells. NGF+ NF now correlated with SCORAD and mast cell-NF contacts with itch in non-lesional skin. At the same time, SLURP-2 levels increased in lesional AD skin.

**Conclusions:**

In humans chronic inflammatory and highly acute psycho-emotional stress interact to modulate cutaneous neuro-immune communication and NNCS marker expression. These findings may have consequences for understanding and treatment of chronic inflammatory diseases in the future.

## Introduction

Animal and human studies over the past two decades provide ample evidence for a significant impact of perceived stress on inflammation in organs at self-environment borders such as the skin [1]. An exemplary chronic inflammatory skin disease, atopic dermatitis (AD), greatly contributed to our understanding of the underlying neuroendocrine-immune interactions [2]–[8]. Most studies investigating the link between heightened stress and worsened inflammation in humans demonstrated a contribution of the hypothalamus pituitary adrenal axis (HPA) and the sympathetic axis (SA) to its characteristic immune profile: prominent production of pro-allergic cytokines such as interleukin (IL)-4 and IL-5, which drive humoral and eosinophilic inflammation and dominate the initial lesion. Later on, cytokines driving cellular adaptive immunity rise in addition, including tumor necrosis factor alpha (TNFα) and interferon gamma (IFNγ).

In addition, a substantial number of studies - mainly done in animal experimental models that utilize inbred mice - have demonstrated the contribution of neurotrophin- and neuropeptide-dependent neurogenic inflammation to stress-induced aggravation of allergic flares [9]–[12]. This innate immune response, accounts for about 50% stress-induced worsening of skin inflammation in mice with an experimentally induced allergic dermatitis [13], [14]. These basic research findings support the concept that maladaptive neuroendocrine-immune interaction is a potent source of stress-induced worsening of AD. However, in humans this remains to be proven [1], [6], [15].

In addition and supporting the development of new pathogenic concepts, the cholinergic system in non-neuronal cells (NNCS) has been identified as a potential player in the skin's response to stress. Stress leads to the release of acetylcholine (ACh) and activation of its associated receptors (AChR) may play an important role in stress-related cytokine imbalance and exacerbation. Accordingly, several authors have shown dysregulation of the cholinergic system in AD and other inflammatory skin diseases, both in the brain and in tissues bordering the environment such as the skin [16]–[20]. Endogenous modulators of AChR known as secreted mammal Ly-6/urokinase-type plasminogen activator receptor-related protein-1 (SLURP-1) and -2 (SLURP-2) now emerge as important ligands of these receptors. Among other targeted nicotinic acetylcholine receptors (nAChR), SLURP-1 was shown to act as an allosteric agonist with high affinity at the α7nAChR enhancing its ACh binding, while SLURP-2 was shown to strongly compete with ACh at the α3nAChR [21]. These proteins regulate immune responses of lymphocytes, dendritic cells and macrophages [22] and suggest themselves as intriguing key players that await analysis in the context of stress.

We here wanted to know if inflammation induced changes in neuro-immune interaction as well as the NNCS occurs in humans and whether they play a role in the skin's response to stress. To follow up on this hypothesis we here employed acute laboratory stress in AD patients as an instructive model to study neuro-immune plasticity and expression of NNCS markers under conditions of acute perceived stress and chronic inflammation. We subjected AD patients and well selected age and sex matched healthy controls to the standardized and validated highly acute Trier Social Stress Test (TSST) and determined neuronal plasticity and mast cell phenotype as well as SLURP and nAChR expression in biopsies at baseline and after stress-exposure. With this approach we found that neuro-immune communication and NNCS mediators do play a role in the complex interdependency between acute stress and chronic inflammation.

## Materials and Methods

### Study-design and ethics votum

The study was approved by the ethics committee of Justus-Liebig University, Giessen, Germany. Participants were recruited using flyers and advertisements. After obtaining written informed consent, participants were enrolled in the study. The participants were informed to take their usual breakfast and avoid coffee, tea and physical exercise on the morning of the stress study. Experiments took place on two consecutive days. During the first day, experiments started at 10.00 h. Upon arrival, participants rested for 30 min then full thickness punch biopsies were taken as described below, followed by another 30 min rest. Participants then received instructions about the TSST and had 15 min for preparation (see description below). After TSST, participants had a final rest of 30 min. During the procedure, 7 times saliva samples and psychometric data were obtained. On the following day (24 h after TSST) a second biopsy per skin area was taken on the contralateral side. This protocol thus required multiple biopsies plus stress exposure. We were therefore highly motivated and instructed by the ethics committee to keep the number of participants required as low as possible and employed rigorous inclusion and exclusion criteria (described below) to reduce variance not related to experimental conditions as much as possible.

### Selection of participants and inclusion and exclusion criteria

Participants older than 18 and younger than 50 years of age were included. AD patients had confirmed diagnosis of AD according to international criteria of Hanifin [23] with duration not less than 1 year and SCORAD score above 30. Exclusion criteria were as follows: Secondary infection or Herpes simplex in the area of biopsy; manifested bronchial asthma or allergic rhinitis; severe mental disorder; severe somatic disorder including obesity; oral intake of drugs and medications with influence on stress and immune response (alcohol, nicotine, drugs, contraceptives, glucocorticoids, ß-adrenergic, antihistaminic or psychotropic medications, cyclosporine A and others); UV-therapy conducted less than one week before experiment; local application of glucocorticoid or immunosuppressive medications in the area of biopsy less than 48 h before experiment. Of the 58 AD patients who applied, 23 could be invited for clinical examination out of which 11 (eight male, three female) matched the criteria. Due to technical issues not all samples could be analyzed in each assay, respective numbers are indicated in the figure legends. Control subjects were matched to patients in age and gender as well as biopsy location for each assay. In addition, they were matched with respect to education, social class and environmental conditions. The resulting study population consisted of educated healthy Caucasians who were either without skin disease or had extrinsic AD with a personal history longer than 1 year.

### Biopsy acquisition

4 mm diameter punch-biopsies were taken under local anesthesia with 1% Scandicain (Mepivacain, AstraZeneca, Wedel, Germany) in localizations typical for AD (mostly elbow flexural side, occasionally knee flexural side, not from head or neck region) 1 h prior to TSST and 24 h later. Matching locations were chosen to obtain biopsies from non-lesional and control skin. After biopsy was taken, the wound was closed with one stitch, which was removed after 7–10 days. Skin samples were immediately frozen in liquid nitrogen and stored at −80°C until further analysis.

### Acute stress exposure: the Trier Social Stress Test (TSST)

The TSST [24] is a standardized and well validated stress paradigm frequently used in psychoneuroimmunology to reliably induce moderate acute psychosocial stress in a laboratory setting. Participants were invited to the laboratory in the morning and after the biopsy and a rest as described above, they were given the instructions to the TSST. They were allowed to prepare a free speech on a novel topic for 15 min in a separate room adjacent to the experimental room. The subjects were then told to enter a brightly lit room without their notes and give the speech (5 min) publicly in front of a microphone, a camera and an expert committee that was to evaluate their performance. The committee consisted of three harsh faced white lab coated interviewers, trained to give no social feedback. After the free speech they were told to do mental arithmetic by giving them a serial subtraction task (5 min). When they paused, subjects were told to continue and to restart when making an error. To control effectiveness of TSST, salivary cortisol levels were measured. Time points for sample acquisition were as follows: 1 =  after rest before biopsy, 2 =  immediately after biopsy, 3 = 30 min after rest following biopsy, 4 =  after instruction and preparation for TSST, 5 =  after TSST, 6 = 10 min after TSST, and 7 = 30 min after TSST.

### Immunofluorescence-histomorphometry

Cryostat sections (10 µm), fixed in acetone (at −20°C, 10 min), were incubated overnight at room temperature with the primary antisera to PGP 9.5 (1∶100, Biotrend, Cologne, Germany), Gap43 (1∶400, EURO-DIAGNOSTICA, Heidelberg, Germany), Nerve growth factor (NGF) (1∶75, SantaCruz, Heidelberg, Germany) or tryptase (1∶200, R&D Systems, Wiesbaden, Germany) as described previously [26]. This was followed by an incubation of 60 min at 37°C with tetramethylrhodamine-isothiocyanate (TRITC)-conjugated F(ab)2 fragments of goat anti-rabbit IgG (Dianova, Hamburg, Germany) diluted 1∶200 in tris-buffered saline. Cell nuclei were counter-stained with 4′,6-diamidino-2-phenylindole dihydrochloride (DAPI, Boehringer Mannheim, Mannheim, Germany, 1 µg/ml, 1 min, room temperature) and mast cells with fluorescein-labeled avidin (FITC-avidin, 1∶2000, Linaris, Wertheim, Germany). Negative controls were incubated with the secondary antibody alone or with a mixture of the primary antibody and the control peptide for the specific anti-serum. Labeling of NF (PGP 9.5, Gap43, NGF) and mast cells (tryptase, FITC-avidin) in full thickness scalp skin biopsies served as internal positive controls. Mast cells were also detected by Giemsa (Merck, Darmstadt, Germany) staining to assess degranulation as described elsewhere [25]. Stained sections were examined at 400x magnification under a Zeiss Axioscope 2 microscope with a daylight as well as fluorescence device (Zeiss, Göttingen, Germany). Each staining pattern was analyzed in the compartments epidermis (epidermal thickness) and upper dermis (NF and mast cells). Histomorphometry was performed by counting NF profiles and mast cells per microscopic field as described before [26]. Each staining-pattern was evaluated in at least five different microscopic fields per analyzed sample and compartment.

### Reverse-transcriptase (RT) PCR

For RT-PCR, skin samples were transferred into lysis buffer (Qiagen, Hilden, Germany) and minced by using an Ultra-Turrax (IKA-Labortechnik, Staufen, Germany). Total RNA was isolated using RNA spin columns according to the manufacturer's recommendations (RNeasy kit, Qiagen). Contaminating DNA was removed using DNase (1 U/Sg total RNA, Gibco-BRL, Life Technologies, Karlsruhe, Germany) in the presence of 20 mM Tris-HCl (pH 8.4), 2 mM MgCl_2_, 50 mM KCl for 15 min at 25°C. Equal amounts of RNA were reverse transcribed with Superscript RNase H- Reverse transcriptase (Gibco-BRL) for 50 min at 42°C. For the PCR reaction, buffer II, 2 mM MgCl_2_, 0.25 mM dNTP (10 mM each), 0.5 U/25 Sl AmpliTaq Gold polymerase (all reagents from Perkin Elmer, Rodgau, Germany) and 20 µmol/lSM of each primer (MWG Biotech, Ebersberg, Germany) were mixed. PCR primers were designed using Primer Express software (Applied Biosystems, Foster City, USA). Primers used for RT-PCR: ß-Actin, forward: gtggcatccacgaaactacctt, reverse: gagtacttgcgctcaggagga, accession no. NM_001101; α3nAChR, forward: cagagtccaaaggctgcaag, reverse: agagagggacagcacagcat, accession no. NM_000743.4; α7nAChR, forward: gtacgctggtttccctttga, reverse: ccactaggtcccattctc, accession no. U40583; SLURP 1, forward: acggtggaggcagagtacc, reverse: ggaagcagcagaagatcagg, accession no. NM_020427.2; SLURP 2, forward: catggatccagatgcctgag, reverse: catatcggggcagcctatgt, accession no. NM_023946.2. Primers for 18S-ribosomal RNA (rRNA) were purchased from Ambion, Huntington, UK. Cycling conditions for PCR were 10 min at 95°C, 40 cycles of 30 s at 94°C, 20 s at 62°C and 30 s at 73°C followed by 7 min at 73°C. RT-PCR products were identified by sequencing (MWG Biotech).

### Quantitative Real-time PCR (qRT-PCR)

qRT-PCR (iCycler, Bio-Rad, München, Germany) was used to quantify levels of α3nAChR and α7nAChR, as well as SLURP-1 and SLURP-2 mRNAs in specimens from lesional and non-lesional AD skin and from healthy control skin. Preparation of cDNA was done as described for RT-PCR. All PCR reactions were prepared in triplicates using a ready-to-use kit according to the manufacturer's protocol (SYBR green, iQ SYBR Green Supermix, Bio-Rad). The data were normalized by subtracting the threshold cycle (CT) levels between the nAChRs/SLURPs and ß-actin. The relative nAChRs/SLURPs expression in the control skin compared to lesional and non-lesional skin was determined by calculating 2^(50-ΔCT lesional skin)-(50–ΔCT non-lesional skin)^.

### Statistics

Relatively low numbers of participants as included here require employment of adequate analytical strategies, especially when multiple biomarkers are assessed. According to the different questions addressed (see results section), we performed mainly pairwise comparisons. In the case of non-matched groups, Mann–Whitney U-test for comparison between two groups was conducted (controls vs non-lesional AD and controls vs lesional AD). In the case of dependent variables (non-lesional AD vs lesional AD and pre-, post-stress) Wilcoxon test was performed. To compare cortisol, pulse and tension values for each measurement (controls vs. AD) T-test was calculated. Intercorrelations between variables were tested by calculating Kendall tau b correlation coefficients. Differences were considered statistically significant when p<0.05.

## Results

### Chronically inflamed skin is associated with higher neuronal density but not neuronal plasticity

**Figure 1:**
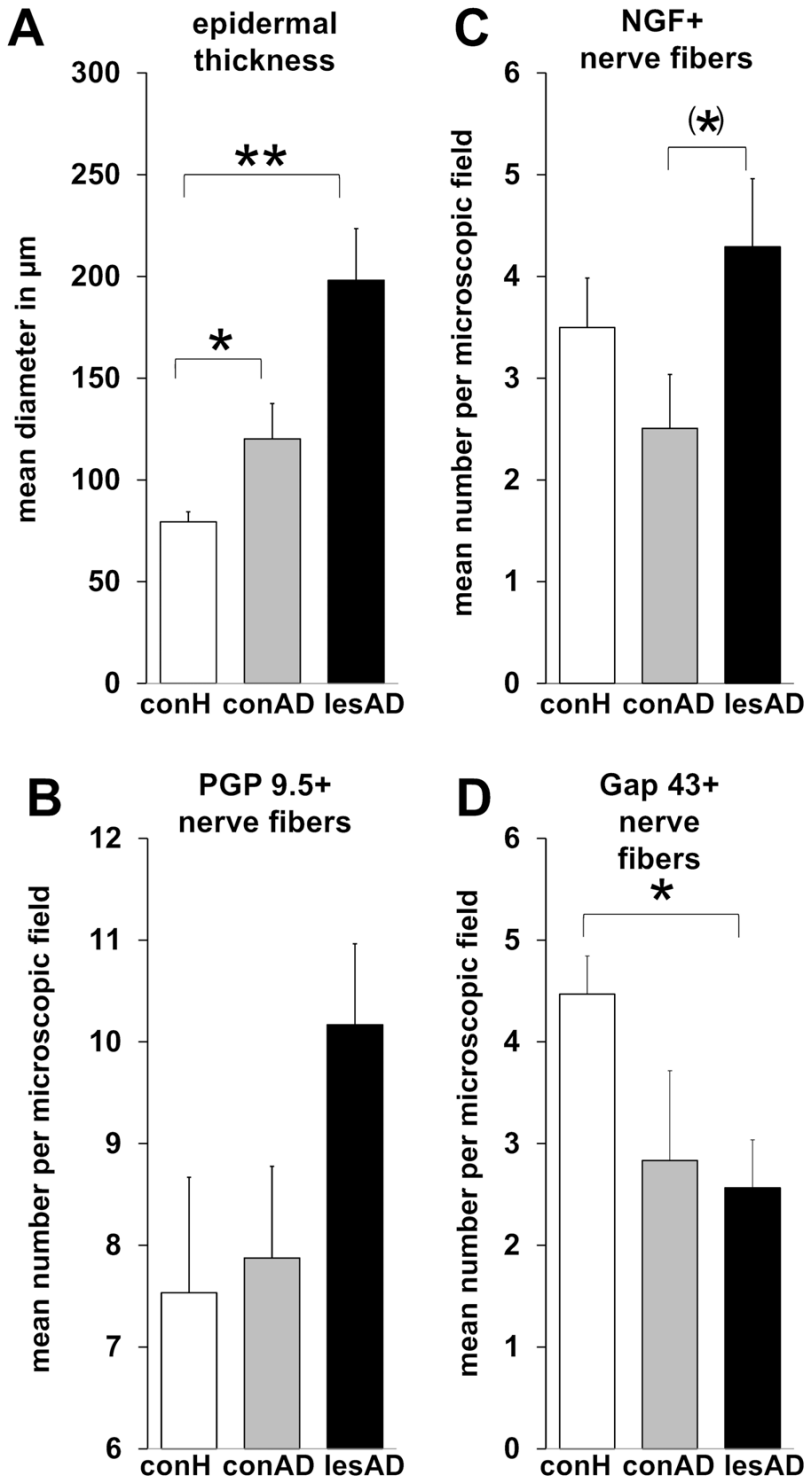
Epidermal thickness and NF in healthy, non-lesional and lesional AD skin. Epidermal thickness and number of IR NF were determined per microscopic field in stained cryo-sections of full thickness skin as described before [26]. Participants per group (control, non-lesional AD, lesional AD): epidermal thickness N = 9, 8, 11; NGF+ NF N = 11, 11, 11; PGP 9.5+ NF N = 4, 5, 6; Gap43+ NF N = 8, 3, 5. Corresponding raw data is provided as **Data S1**. P-values: <0.10 =  (*), <0.05 = *, <0.01 = **. Abbreviations: conH – healthy control skin, conAD – non-lesional AD skin, lesAD – lesional AD skin.

First we wanted to know if analogous to the mouse model allergic inflammation alters neuronal density and plasticity in human skin. Presence of chronic inflammation in AD skin was confirmed by altered epidermal thickness (**Figure 1A**). At baseline, lesional skin of AD patients compared to healthy control skin showed higher numbers of PGP 9.5+ NF profiles in the subepidermal upper dermis (**Figure 1B**), a key cutaneous compartment for neuro-immune interaction between NF and mast cells. This was accompanied by higher NGF detection in upper dermal NF in lesional AD skin (**Figure 1C**). By contrast we found significantly lower numbers of Gap43-immunoreactive NF, a marker for growing NF, in the upper dermis of lesional AD skin (**Figure 1D**). Assessment of correlations employing Kendall tau b revealed a significant positive correlation between epidermal thickness and NGF+ NF (tau 0.600, p = 0.045) and a significant positive correlation between SCORAD and NF-mast cell contacts in lesional skin (tau 0.894, p = 0.008) as well as between itch and NGF+ NF in non-lesional (tau 0.466, p = 0.028) and itch and NF-mast cell contacts (tau 0.745, p = 0.022) in lesional AD skin. Finally we observed a negative correlation between itch and degranulated mast cells (tau −0.800, p = 0.025).

### AD skin lacks mRNA for α7nAChR and its ligand SLURP-1

**Figure 2:**
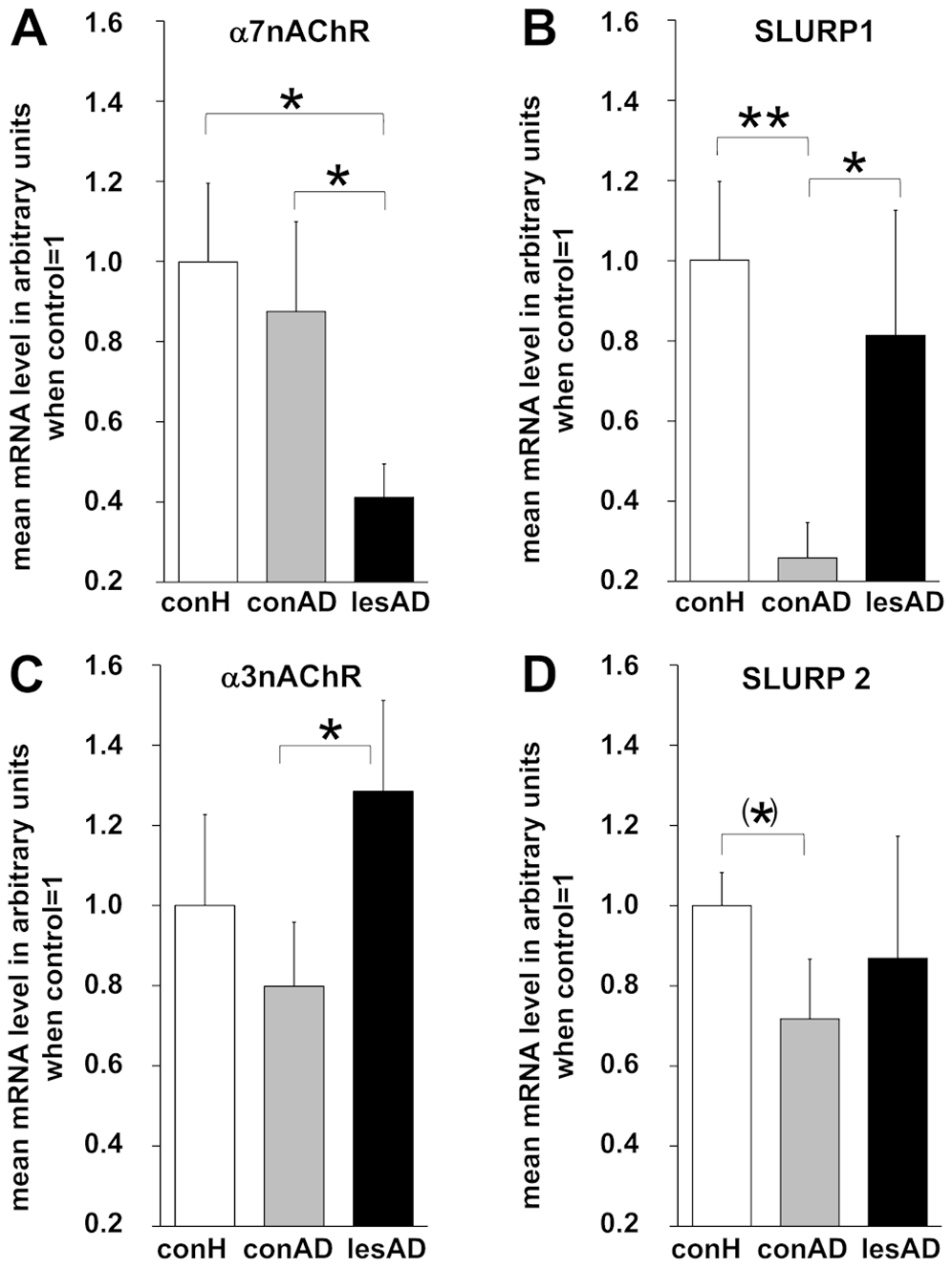
SLURP and selected SLURP receptor levels in healthy, non-lesional and lesional AD skin. Quantitative RT-PCR was performed on full thickness skin biopsies. Participants per group: eight (six male, two female). Corresponding raw data is provided as **Data S1**. P-values: <0.10 = (*), <0.05 = *, <0.01 = **. Abbreviations: conH – healthy control skin, conAD – non-lesional AD skin, lesAD – lesional AD skin.

Next we wanted to know if NNCS marker expression differed between healthy and inflamed human skin. α7nAChR mRNA in full thickness skin biopsies was lowest in lesional AD skin while mRNA level for its endogenous ligand SLURP-1 was lowest in non-lesional AD skin (**Figure 2A and B**). α3nAChR and SLURP-2 showed the same expression pattern as SLURP-1 though less pronounced (**Figure 2C and D**). Assessment of correlations employing Kendall tau b revealed a highly significant positive correlation between α7nAChR mRNA and SLURP-1 and -2 levels (tau 0.857, p = 0.001; tau 0.786, p = 0.003).

### Acute stress provoked hypothalamus pituitary adrenal (HPA) activation, increased heart rate (SA) and perceived tension irrespective of skin inflammation

Thirdly, we wanted to know if the TSST was performed effectively to induce acute stress and whether we could reproduce the published data on altered HPA reactivity in AD patients. The stress response was monitored by measuring salivary cortisol level (HPA), pulse (beats per min, SA) and perception of tension (Likert scale) throughout the experiment. We also monitored the stress response during the taking of the biopsy prior to TSST. Both groups mounted a rise in the assessed parameters in response to the TSST. AD patients in contrast to controls responded to the taking of the biopsy (time point 2) with a small though not significant cortisol rise and pulse drop. Both groups reported increased perceived tension at time point two. Hence the taking of the biopsy increases stress perception with a small but not significant HPA and SA response in AD patients but no HPA and SA response in healthy controls. AD patients also reported significantly higher perception of tension at baseline and at time points preceding the TSST (3 - rest after biopsy preceding preparation for TSST, 4 – after TSST instruction and preparation). At these time points however cortisol levels did not differ from controls and both groups mounted a strong response to the TSST indicating that the taking of the biopsy did not attenuate the stress response generated by the TSST.

**Figure 3:**
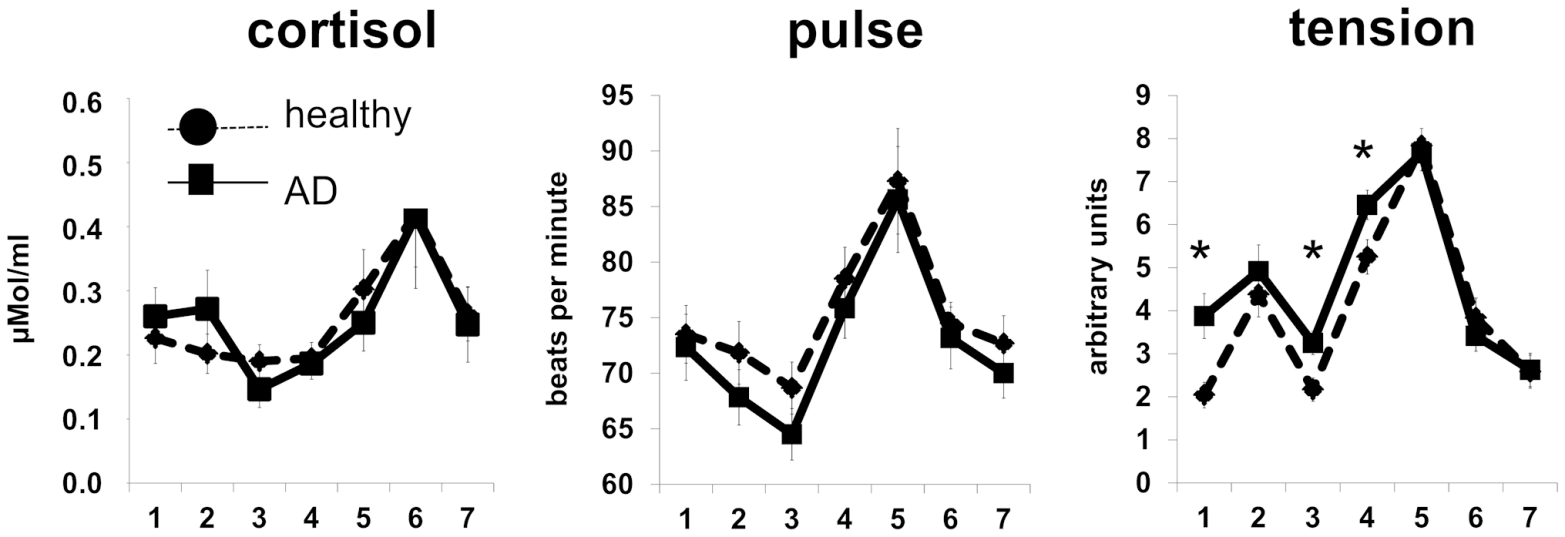
Stress response during TSST. During TSST salivary cortisol levels (HPA), pulse (beats per min, SA) and tension assessment (Likert scale, psychoemotional stress perception) were obtained 7 times. Time points: 1 =  after rest before biopsy, 2 =  immediately after biopsy, 3 = 30 min after rest following biopsy, 4 =  after instruction and preparation for TSST, 5 =  after TSST, 6 = 10 min after TSST, 7 = 30 min after TSST. Corresponding raw data is provided as **Data S1**. P-values: <0.05 = *.

Our results demonstrated that the TSST was effectively performed and not affected by the taking of the biopsy (**Figure 3**). In contrast to the literature, we could not detect a difference in the maximal systemic stress responses between controls and AD patients. Also, the test did not differentiate between men and women included in this study (not shown). However, this may indicate that being mostly male and having a rather mild AD, the patients included here did not serve to demonstrate a systemic alteration of stress axis function by AD (**Figure 3**). Hence, the here reported effects in skin are most likely due to a local response to the TSST.

### Acute stress decreases NF density in lesional AD skin

**Figure 4:**
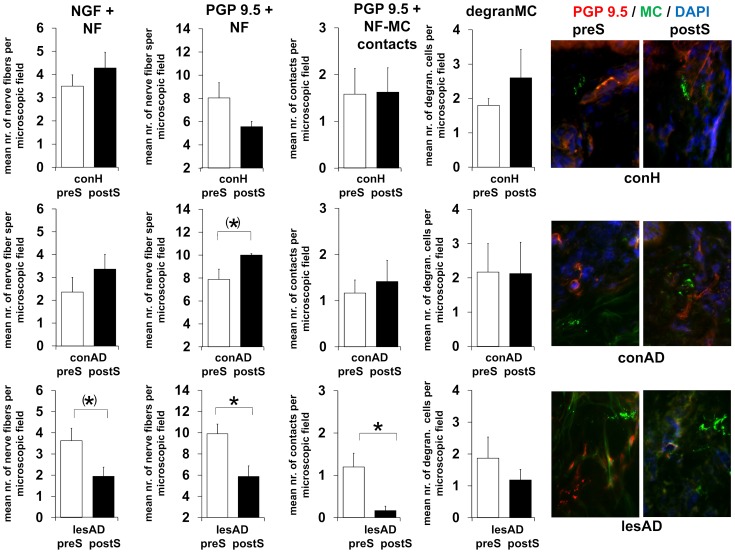
TSST induced neuro-immune changes in healthy control, non-lesional and lesional AD skin. Changes to baseline obtained as described in Figure 1 were determined 24 h after TSST. Number of participants per evaluation (NGF+ NF, PGP 9.5+ NF, PGP 9.5+ NF-mast cell contacts, degranulated mast cells [MC]): control: N = 11, 4, 4, 4; non-lesional AD: N = 11, 4, 4, 4; lesional AD: N = 11, 6, 6, 5. Corresponding raw data is provided as **Data S1**. P-values: <0.10 = (*), <0.05 = *. Abbreviations: conH – healthy control skin, conAD – non-lesional AD skin, lesAD – lesional AD skin, preS – prior to TSST, postS – 24 h after TSST.

To assess if stress alters neuronal density, neuronal plasticity and neuro-immune interaction in human skin was our next aim. We found that exposure to the highly acute TSST reduced the number of detectable NGF+ dermal NF in lesional AD patient's skin along with a decreased PGP 9.5+ NF number and a decreased number of neuro-immune contacts between PGP 9.5+ NF and tryptase+ mast cells in the upper dermis of lesional AD skin (**Figure 4**), while total mast cell numbers remained relatively stable (not shown). Mast cells also showed decreased degranulation, albeit not significantly. Interestingly, non-lesional AD skin showed an opposite tendency with increased numbers NGF+ NF and PGP 9.5+ NF (**Figure 4**). Assessment of correlations employing Kendall tau b revealed a highly significant negative correlation between SCORAD and NGF+ NF (tau -0.539, p = 0.016) and a significant positive correlation between SCORAD and NF-mast cell contacts in non-lesional skin (tau 0.733, p = 0.019). Moreover, we found a significant positive correlation between itch and NF-mast cell contacts in non-lesional skin (tau 0.600, p = 0.045).

### Stress improves SLURP expression in inflamed AD skin

**Figure 5:**
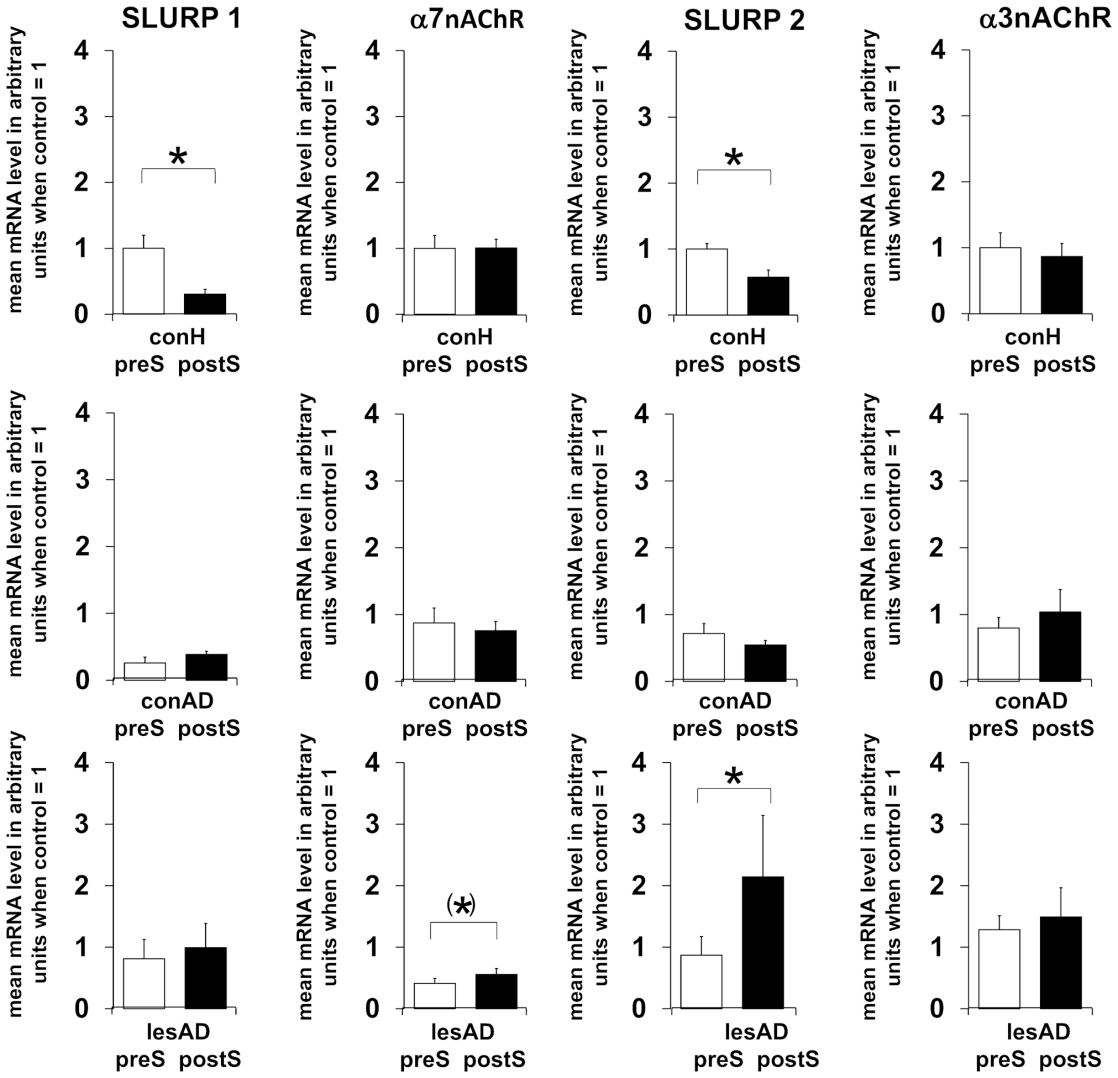
TSST induced changes in SLURP and SLURP receptor levels in healthy, non-lesional and lesional AD skin. Changes to baseline as given in Figure 2 were determined 24 h after TSST. Participants per group: eight (six male, two female). Corresponding raw data is provided as **Data S1**. P-values: <0.10 = (*), <0.05 = *. Abbreviations: conH – healthy control skin, conAD – non-lesional AD skin, lesAD – lesional AD skin, preS – prior to TSST, postS – 24 h after TSST.

Finally we wanted to know if stress affects the cutaneous NNCS in healthy and inflamed human skin. At the mRNA level, expression of SLURP-1 and -2 decreased significantly in healthy skin after TSST (**Figure 5**). In non-lesional skin no changes were provoked by TSST with respect to the investigated NNCS markers. By contrast, in lesional AD skin SLURP-2 increased significantly (**Figure 5**). α7nAChR mRNA also minimally increased after stress in lesional AD skin (**Figure 5**). Assessment of correlations employing Kendall tau b revealed a significant negative correlation between SCORAD and SLURP-2 in non-lesional skin (tau −0.643, p = 0.013).

## Discussion

We here investigated neuro-immune communication as well as NNCS marker expression in non-lesional and lesional AD skin compared to healthy control skin at baseline and post TSST. Multiple biomarkers and data sets were required to allow assessment of these different aspects of neuro-immune interaction as well as NNCS modulation in stress aggravated inflammatory disease. Our first data set demonstrates altered neuro-anatomy in lesional AD skin compared to control in humans which is suggestive for increased neurogenic inflammation. The second data set suggests decreased presence of the investigated potentially anti-inflammatory NNCS markers in AD skin. The third data set demonstrates that after psychosocial stress (TSST) neuro-anatomy in uninflamed skin is significantly changed in a way that facilitates neurogenic inflammation. And finally, the fourth data set demonstrates that NNCS markers are decreased in healthy skin after TSST but increased in inflamed AD skin suggesting a role in the fine tuning of proliferative and inflammatory responses in the skin's response to stress.

Discussing neuro-immune interaction at baseline in detail, an increase in NGF expression and in NF density can be observed in chronically inflamed skin and allergy [14], [27], [28]. We here confirm these results in humans under laboratory stress. In our hands however, increased innervation in chronically inflamed AD skin did not seem to be associated with active growth of NF since we observed lower numbers of Gap43+ nerve fibers in AD skin under steady state conditions. Instead, increasing numbers of NGF+ NF in inflamed AD skin correlated with epidermal thickness, which is a sign of chronic inflammation and a major source of peripheral NGF. Since NGF is taken up by NF and retrogradely transported to the cell body, where it promotes their survival [29], we conclude that enhanced innervation is due to enhanced neurotrophin support and maintenance of existing NF rather than NF growth.

Discussing the role of the NNCS in AD, ACh synthesis and transport was shown to be decreased in allergically inflamed lungs [30] and nicotine has an anti-inflammatory effect in a model of allergic asthma [19]. Genetic deficiency in SLURP-1 leads to defects in keratinocyte differentiation and immune dysfunction, resulting in Mal de Maleda, a skin disease resembling AD of the hands and feet [31]. SLURP-1 was also shown to downregulate TNFα and upregulate NF-kappa B e.g. in asthmatic lung epithelia [32]–[34]. Moreover, inflammatory processes that are characterized by strong innate and cellular immune responses such as eosinophilia and neutrophilia or viral infection are characterized by a lack of SLURP-1 [35], [36]. These processes can also be observed in AD and SLURP-1 therefore appears to control a wide spectrum of AD-relevant immune responses. Taking these known functions of nAChR and SLURPs into account, the absence of NNCS markers in non-lesional AD skin observed here and specifically the lack of α7nAChR in lesional AD skin may relate to the thickened epidermis characterized by dysfunctional cell differentiation [37] and to a shortage of anti-inflammatory effects normally provided by the NNCS.

With respect to the effect of psychosocial stress on non-inflamed versus inflamed skin our results are suggestive for an enhanced neuro-immune communication as early as 24 h after TSST in healthy and in non-lesional AD skin, which can promote neurogenic inflammation in heretofore uninflamed skin. By contrast we observed reduced innervation in lesional AD skin. To understand this observation, it is interesting to note that under highly acute and combined stress conditions, nerve terminals and mast cells are forced to release stress- and inflammatory mediators so rapidly, that even degeneration of peptidergic nerve endings and mast cell apoptosis were described [13], [38]. The decreased number of NF in lesional AD skin after stress observed here may thus be the result of their deleterious activation and degranulation. However, electron microscopy would be required to confirm this idea.

The most interesting finding to us however was the observation that highly acute stress leads to decreased SLURP-1 and -2 mRNA levels in healthy skin while SLURP-2 transcript is increased in inflamed AD skin. Highly acute stress was repeatedly reported to promote a cytokine profile also described as the T-helper cell type 1 (TH1) profile consisting of TNFα and other cytokines that promote adaptive cellular immunity under conditions not involving additional stressors or pre-existing pathologies [1], [6], [39]. A drop in SLURPs in the skin of healthy individuals under acute psychosocial stress could be involved in this reported shift. On the contrary, in skin already subjected to chronic allergic inflammation, a shift of the immune balance towards increased TH2, dominated by pro-allergic cytokines such as IL-4 and IL-5 is reported in response to additional stressors [1], [6], [39]. The SLURP-2 rise in lesional AD skin after acute stress exposure may contribute to this profile.

Finally, to employ few but well matched participants with regard to ethic efforts allowed us to analyze skin biopsies and comprehend the local effects of stress in peripheral tissues while most comparable human studies were restricted to neuroendocrine-immune analysis in blood. Naturally, the results presented here have to be confirmed in larger and more diverse populations. Follow up studies should also recapitulate cellular and molecular mechanisms involved utilizing *ex vivo* models such as skin organ culture. However, this pilot study is instructive and effectively confirms in humans the involvement of neuroendocrine-immune interaction in stress-induced aggravation of allergic inflammation reported in mice. It also demonstrates for the first time to our knowledge the participation of the NNCS and SLURPs in the skin stress response.

## Conclusions

In summary, neuro-immune interaction participates in the pathogenesis and stress-sensitivity of AD in humans. To our knowledge this is also the first report on SLURP and nAChR expression to be altered by acute stress in a life situation in healthy and inflamed tissue. Our results confirm the idea that a steady state of ACh-levels keeps inflammation in general at bay, while under stress modulation of ACh presence and signaling can be altered both by inflammatory as well as by psychoemotional stress [40]. These observations support the validity of the two hit model for stress-immune interaction in disease development and progression and that different stressors and stress-response systems interact to mold a specific response [8], [41]. Further investigations are needed to assess the value of our findings for the initiation and interpretation of stress-reducing therapeutic interventions.

## Supporting Information

Data S1 **Microsoft Excel (xls) file contains all raw data used for statistical evaluation and presented in the results section and figures throughout the paper.**
(XLSX)
